# Diatomite-Mediated Humification and Fungal Community Succession During Composting

**DOI:** 10.3390/microorganisms14061245

**Published:** 2026-06-01

**Authors:** Jun Xie, Min Liu, Xiaoying Mu, Yaxuan Liu, Shaoyan Ma, Yerong Guo, Jiabin Hu, Yuanyuan Wang, Haisheng Yan, Xiaodong Zhao, Yanbo Wang

**Affiliations:** 1 College of Biological Sciences and Technology, Taiyuan Normal University, Jinzhong 030619, China; xj123@tynu.edu.cn (J.X.); lm15698354586@163.com (M.L.); 19834556514@163.com (X.M.); 15603447496@163.com (Y.L.); 13044453370@163.com (S.M.); 15135728029@163.com (Y.G.); fraudname@163.com (J.H.); 2 Shanxi Key Laboratory of Earth Surface Processes and Resource Ecology Security in Fenhe River Basin, Taiyuan Normal University, Jinzhong 030619, China; 3 School of Agriculture, Food and Wine, Waite Research Institute, Adelaide University, Glen Osmond, SA 5064, Australia; yuanyuan.wang01@adelaide.edu.au; 4 Sorghum Research Institute, Shanxi Agricultural University, Jinzhong 030810, China; yanhaisheng@sxau.edu.cn; 5 School of Science, Western Sydney University, Penrith, NSW 2751, Australia

**Keywords:** aerobic composting, pig manure, diatomite, humification, fungal community

## Abstract

Organic-matter degradation and humification degree are key determinants of compost quality. In this study, we used pig manure and wheat straw to investigate the effects of diatomite on organic-component degradation, humification, and fungal community succession. In a 40-day aerobic composting experiment, we compared a control treatment with treatments supplemented with 4% and 8% diatomite. The results indicated that diatomite significantly accelerated organic-matter degradation and humification, with 8% diatomite increasing organic matter and lignin degradation by 9.05% and 9.27%, respectively. Based on linear interpolation of the HA/FA ratio dynamics, it was estimated that the maturity threshold (a ratio of humic acid to fulvic acid > 1.6) was reached 5–7 days earlier in the group subjected to 8% diatomite treatment relative to the control. Fungal community analysis revealed that the 8% diatomite treatment effectively alleviated fungal suppression under high-temperature conditions. By the maturation phase, fungal richness and diversity in the group subjected to the 8% diatomite treatment reached 1.8 and 2.6 times that of the control, respectively, significantly promoting the colonization and recovery of thermophilic Ascomycota, including *Mycothermus* and *Aspergillus*. Diatomite shifted fungal interactions from competition to symbiotic cooperation centered on *Mycothermus*, with partial least squares path modeling confirming fungal composition was a primary regulator of humification. This study demonstrates that 8% diatomite enhances composting efficiency and humification quality by optimizing fungal community structure and function, offering a theoretical and practical basis for the use of agricultural waste resources.

## 1. Introduction

With the continuous increase in the amount of solid waste generated worldwide, using agricultural waste has become a key means of fulfilling the United Nations’ sustainable development goals and alleviating environmental pressure [1]. According to the “Global Waste Management Outlook 2024” report, global solid waste generation is projected to reach 3.8 billion tons by 2050 [2]. Currently, less than 20% of global solid waste is recycled annually, a figure falling short of the requirements for sustainable development [3]. An effective strategy for treating agricultural waste, composting can stably transform organic matter into humus through microbial metabolism [4] and offers significant advantages in the collaborative treatment of livestock and poultry manure and agricultural straw. However, the composting process is slow, primarily because of the low microbial biodegradation rate of complex organic matter (such as cellulose and lignin), not only prolonging the decomposition period but also limiting the degree to which the humus content of compost products can be improved [5]. Therefore, enhancing the degradation of organic matter and promoting humus formation during pig manure–wheat straw composting have become central challenges in the context of improving composting efficiency.

To improve the efficiency of composting, exogenous regulators can be added during production to promote the growth and activity of key microorganisms [6]. Physical regulators such as biochar and zeolite have been extensively investigated [7,8]. These materials can enhance the quality and efficiency of compost by improving the physical structure of the composting matrix and regulating water and gas exchange. As a natural siliceous mineral, diatomite has a highly porous structure and a large specific surface area [9]. During the composting process, it can modulate the fungal microenvironment and promote the growth of fungi by improving the pore structure, regulating the exchange of water and gas [10], and adsorbing potential inhibitory substances (such as ammonia and organic acids), showing unique potential in composting. Although previous studies have investigated the role of diatomite in mitigating nitrogen loss and suppressing the transfer of antibiotic resistance genes during composting [11], they primarily focused on pollutant control and the effects of such amendments on the physicochemical properties of and bacterial communities in compost [12], while the mechanisms by which diatomite drives the efficient degradation of lignocellulose remain largely unexplored.

During the degradation of lignocellulose in compost, enzymes secreted by the fungal community directly determine the transformation pathways and efficiency of the organic components in the compost [13]. In contrast to bacteria, fungi can not only withstand extreme environmental conditions (such as high pH and temperatures) [14] but also produce a suite of extracellular enzymes that enhance the mineralization of complex organic matter in composting feedstocks. Consequently, they are considered indispensable drivers of the composting process [15]. Filamentous fungi, particularly members of Ascomycota and Basidiomycota, play a dominant role in the degradation of lignocellulosic biomass (e.g., wheat straw) by secreting a diverse suite of carbohydrate-active enzymes. These include glycoside hydrolases (e.g., cellulases and hemicellulases) and auxiliary activity enzymes (e.g., lignin-modifying peroxidases and laccases) [16,17,18]. Fungi directly decompose the recalcitrant lignin framework, whereas bacteria exhibit limited efficiency in breaking down the lignocellulose core [19]. Therefore, investigating the influence of diatomite during composting from a fungal community perspective can foster a more direct understanding of the microbiological mechanisms underlying enhanced organic matter transformation. Based on these insights, we hypothesized that diatomite amendment can optimize the composting microenvironment, thereby steering fungal community succession toward a highly cooperative network dominated by key thermophilic functional taxa, ultimately driving efficient lignocellulose degradation and deep humification.

To elucidate the effect of diatomite on composting efficiency and the underlying microbiological mechanism, we established a control group (CK), which was not treated with diatomite, and two treatment groups treated with 4% (DE4) and 8% (DE8) diatomite, respectively. The degree of humification, the degradation dynamics of organic components (e.g., lignocellulose and organic matter), and fungal community succession were compared between the groups. The results of this study are expected to provide theoretical support and practical reference value for optimizing the composting process of organic waste and enhancing the efficiency of mineral amendments in waste resource utilization.

## 2. Materials and Methods

### 2.1. Experimental Materials and Setup

The raw materials for the experiment, pig manure, was collected from a medium-sized farm in Yangling District, Xianyang City, Shaanxi Province, China. Wheat straw was collected from a local farm, and diatomite was purchased from an ore factory in Lingshou County, Hebei Province. Composting experiments were conducted in a laboratory-scale composting reactor (with a working volume of approximately 70 L). The reactors were placed in a greenhouse in Yangling and the ambient temperature during the composting period was monitored daily using a digital thermohydrometer, exhibiting an average of 21.5 ± 3.2 °C. The pig manure and wheat straw were mixed well, the initial C/N ratio was adjusted to 26: 1, and the initial water content (MC) was kept at about 60% through the addition of distilled water. Three treatment groups were established: a control group with no diatomite treatment (CK) and the treatment groups with diatomite added at 4% and 8% on a dry-weight basis ((*w*/*w*) that is, the mass of diatomite relative to the total dry mass of the initial pig manure–wheat straw mixture), designated as DE4 and DE8, respectively. The proportion of diatomite was determined based on previously reported effectiveness in pollutant reduction [20] and a preliminary experiment on composting-microenvironment optimization. Each treatment was set up in triplicate (with three identical reactors per treatment, giving a total of nine reactors) to ensure statistical reliability. To ensure sufficient oxygen supply, intermittent forced aeration was applied from the bottom of the reactors using an air compressor. Aeration was controlled by a time relay and set to run for 5 min every hour, with an aeration rate of 0.35 L/kg (DW)/h during the working period.

### 2.2. Composting Process and Sample Collection

The composting experiment was conducted for 40 days until the compost temperature dropped to room temperature and remained stable. Compost samples were collected on days 0, 3, 9, 14 and 40, representing different temperature-defined stages (Table 1). There were three replicates for each treatment. To obtain a representative sample, the compost was thoroughly turned before 500 g of composite material was collected from each treatment replicate through multi-point sampling and homogenization. The collected samples were divided into two parts: one part was stored at 4 °C for physical and chemical analysis, and the other part was snap-frozen in liquid nitrogen and then stored at −80 °C for DNA extraction.

### 2.3. Determination of Physicochemical Properties and DNA Extraction

Pile temperature and ambient temperature were recorded daily at 3:00 p.m. using a thermometer. Organic-matter content was measured via the potassium dichromate external heating oxidation–colorimetric method. Lignin, cellulose, and hemicellulose content were analyzed according to the Van Soest detergent fiber analysis procedure [21]. Humic and fulvic acids were extracted from plant materials using a modified humus composition method, and the content of each one was measured using the potassium dichromate external heating method [22]. Total genomic DNA was extracted from 0.100 g of compost samples stored at −80 °C using the Fast DNA SPIN Kit for Soil (MP Biomedicals, Santa Ana, CA, USA), which was used as per the manufacturer‘s instructions. The concentration and purity of DNA were determined using NanoDrop 2000 (Thermo Scientific, Wilmington, DE, USA). The results showed that the DNA concentrations ranged from 45.3 to 193.7 ng/µL, with A_260_/A_280_ ratios of 1.82–1.90, ensuring there were high-quality templates for subsequent analysis. All qualified DNA samples were diluted to uniform concentrations and sent to Shanghai Majorbio Bio-Pharm Technology Co., Ltd. (Shanghai, China), for Illumina MiSeq high-throughput sequencing. To characterize the fungal community succession, the fungal internal transcribed spacer 1 (ITS1) region was amplified using the specific primers ITS1F (5′-CTTGGTCATTTAGAGGAAGTAA-3′) and ITS2R (5′-GCTGCGTTCTTCATCGATGC-3′). After PCR amplification and product purification, sequencing libraries were constructed using the TruSeq Nano DNA Sample Preparation Kit (Illumina, San Diego, CA, USA) as per the manufacturer’s technical guidelines, which also involved the ligation of sequencing adapters. The quality and concentration of the resulting libraries were assessed using a Bioanalyzer (Agilent 2100, Santa Clara, CA, USA) and the Quant-iT™ PicoGreen^®^ dsDNA Assay Kit. Finally, the prepared libraries were sequenced on an Illumina MiSeq platform in paired-end mode (2 × 300 bp) utilizing the MiSeq Reagent Kit v3 (Illumina).

### 2.4. Data Analysis

The dynamic curves of key physical and chemical parameters during composting were plotted with Origin software 2021 version. All statistical tests, including one-way analysis of variance (ANOVA), Duncan’s multiple range test, the Shapiro–Wilk test, and Levene’s test, were performed in R (v4.2.0). Statistical significance was determined via one-way analysis of variance (ANOVA) followed by Duncan’s multiple range test (*p* < 0.05) to handle multiple comparisons between different treatments. Before ANOVA was performed, the normality of the data was evaluated using the Shapiro–Wilk test, and the homogeneity of variances was verified using Levene’s test. All data were deemed to have met these assumptions (*p* > 0.05 for both tests) prior to further analysis. To account for variations in sequencing depth, the fungal OTU/ASV table was normalized by rarefying each sample to the minimum sequencing depth prior to downstream bioinformatic analyses. Microbial bioinformatics, including fungal alpha-diversity (e.g., ACE and Chao1 indices) and community structure analysis (PCoA based on Bray–Curtis distance), were employed using Python (v3.8) and R (v4.2.0) with the vegan and phyloseq packages. The detailed analysis pipeline and parameter configurations strictly followed the established methodology described by Darriaut et al. [23]. TBtools (v1.09) was utilized to generate heatmaps of the top 45 fungal genera, with relative abundance data subjected to Z-score normalization to standardize effects across taxa. The fungal co-occurrence network was constructed based on Spearman correlation calculated in R (v4.2.0). To ensure network robustness, only strong and significant correlations (|r| > 0.6 and *p* < 0.05) were retained. Network topological metrics, such as average degree, density, and modularity, were calculated and visualized in Gephi (v0.9.2). A partial least squares path model (PLS-PM) was implemented using the plspm package in R (v4.2.0) to quantify the causal relationships between lignocellulose, OM, fungal community composition, and humification degree. The model’s validity was assessed using the Goodness of Fit (GoF) index, while the reliability of latent constructs was verified by checking that outer loadings exceeded 0.7. The total effects were decomposed into direct and indirect paths, with significance determined via bootstrapping (1000 resampling instances).

## 3. Results

### 3.1. Effect of Diatomite on the Degradation of Organic Components

**Figure 1:**
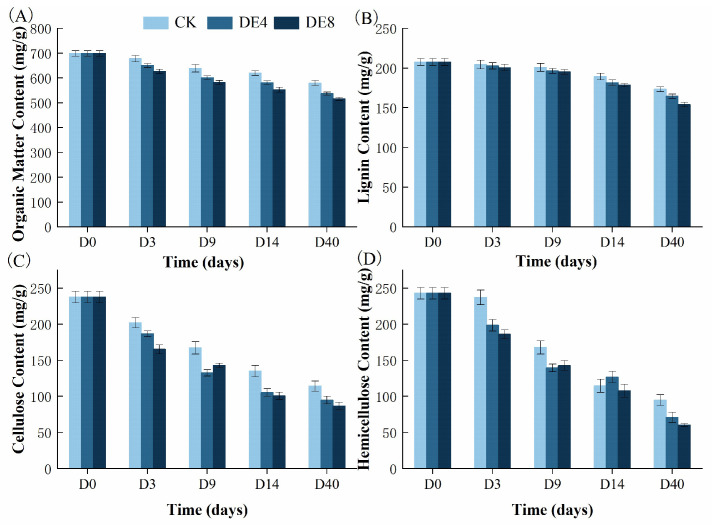
The effects of diatomite addition on dynamic degradation of organic components during composting: (**A**) organic-matter content, (**B**) lignin content, (**C**) cellulose content, and (**D**) hemicellulose content. CK treatment (control), DE4 treatment (4% diatomite), and DE8 treatment (8% diatomite). Sampling time points: D0 (day 0), D3 (day 3), D9 (day 9), D14 (day 14), and D40 (day 40). The whiskers on the graphs represent the standard deviation (SD) of triplicate samples (*n* = 3).

Organic-matter content serves as a crucial index for evaluating the quality of compost products [24]. Figure 1A shows that the organic-matter content in all treatments exhibited different degrees of reduction during composting. During the thermophilic phase (3–14 days), organic-matter degradation accelerated, resulting in organic-matter content reductions of 8.54%, 10.64%, and 11.75% in the CK, DE4, and DE8 treatments, respectively. This sharp decrease was likely associated with the intensified metabolic activity of microorganisms during the thermophilic phase, which potentially accelerated the decomposition of organic matter. The notably lower organic matter content in DE4 and DE8 in comparison with the CK treatment suggests that diatomite addition may promote the degradation of organic matter. Over the 40-day composting period, organic matter content decreased by 17.16%, 23.13%, and 26.21% compared with those in the initial stages in the CK, DE4, and DE8, respectively. This phenomenon suggests that diatomite likely modulates the microenvironment of compost piles, thereby potentially helping to create more-stable conditions for the fungal degradation of organic matter.

Figure 1B shows that there were few differences in lignin content between treatments at the start of composting. Throughout the thermophilic phase (3–14 days), lignin degradation appeared to be enhanced by diatomite amendment with degradation rates of 7.44%, 10.32%, and 11.04% in the CK, DE4, and DE8, respectively. The maturation phase (14–40 days) constituted the principal stage for lignin breakdown, accounting for additional decreases of 8.51%, 9.45%, and 13.72% in the CK, DE4, and DE8, respectively, relative to the values on day 14. The slow degradation of lignin in the CK was likely associated with the inferior microenvironmental conditions (including with respect to aeration and water retention capacity), which potentially suppressed the metabolic activity of the fungal community. Conversely, diatomite amendment potentially improved the microenvironment, thereby contributing to the creation of more stable living conditions for lignin-degrading bacteria. By the end of composting process, the cumulative lignin degradation rates in the DE4 and DE8 were 4.18% and 9.27% higher than the rate in the CK, respectively. This result suggests that diatomite had a long-term promotive effect on the degradation of lignin, which could be associated with diatomite’s ability to improve pile aeration, maintain an optimal temperature, and provide aerobic metabolic conditions for lignin-degrading bacteria.

Similar to the trends observed for lignin, the decomposition of cellulose and hemicellulose was also notably enhanced by diatomite amendment. As illustrated in Figure 1C,D, cellulose and hemicellulose content decreased significantly (*p* < 0.05) throughout composting. Over the 40-day composting period, cellulose and hemicellulose degradation rates reached 51.94% and 61.02% in the CK, 60.08% and 70.78% in DE4, and 63.52% and 75.22% in DE8, respectively. The addition of diatomite was associated with enhanced degradation of cellulose and hemicellulose. This effect could be due to diatomite’s high adsorption capacity and abundant surface functional groups, which likely provided a suitable habitat microenvironment for fungi. Such favorable conditions may promote the colonization and active metabolic expression of thermophilic degrading bacteria, thereby potentially prolonging the thermophilic phase and accelerating the decomposition of cellulose and hemicellulose.

### 3.2. Effect of Diatomite on the Humification of Compost

**Figure 2:**
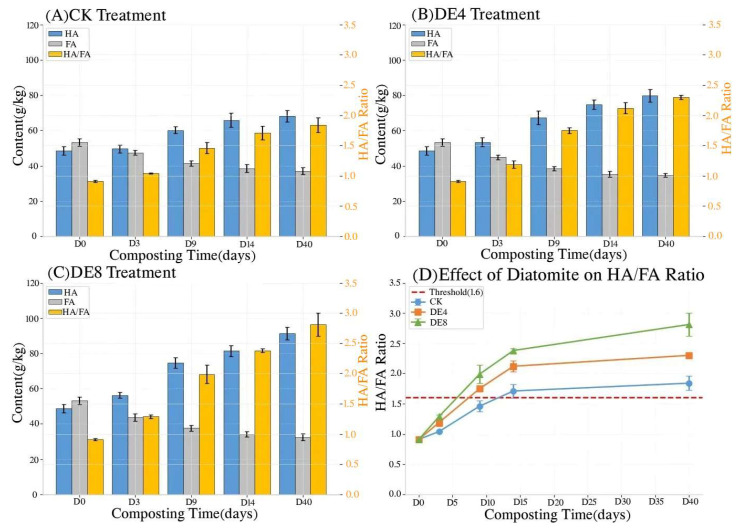
Dynamic evolution of humic substances and maturity indices during composting amended with diatomite. (**A**) CK treatment (control), (**B**) DE4 treatment (4% diatomite), and (**C**) DE8 treatment (8% diatomite), illustrating the concentrations of humic acid (HA, g/kg) and fulvic acid (FA, g/kg) and the corresponding HA/FA ratio. (**D**) Comparative analysis of the HA/FA ratio across all treatments during the composting process; the red dashed line represents the maturity threshold (with a reference value of 1.6). Whiskers in the graphs represent the standard deviation (SD) of triplicate samples (*n* = 3).

In this study, the humic acid (HA) content in each treatment group exhibited a progressive increase throughout the composting process (Figure 2A–C). As composting progressed, HA content gradually increased, reaching 68.12 g/kg, 79.80 g/kg, and 91.37 g/kg at the maturity stage (day 40), with the most pronounced enhancement observed in DE8. From a dynamic perspective, the HA content in DE4 and DE8 exceeded that in the CK during the late thermophilic phase (day 9) and cooling phase (day 14), suggesting that diatomite addition accelerated the synthesis and accumulation of HA. Concurrently, fulvic acid (FA) content demonstrated a gradual decline (Figure 2A–C). The initial FA concentration in the CK, DE4, and DE8 was 53.18 g/kg, which decreased to 37.01 g/kg, 34.67 g/kg, and 32.49 g/kg at the maturity stage, respectively. The most substantial reduction in FA was observed in DE8, implying that diatomite addition markedly promoted the degradation and transformation of FA. Compared with the CK, the diatomite-amended groups exhibited a more rapid HA accumulation rate following the thermophilic phase and a markedly elevated content at maturity, following a clear dose–response relationship: DE8 > DE4 > CK. The FA degradation rate also increased with diatomite dosage, further indicating that diatomite may effectively facilitate the transformation of FA into HA and contribute to the structural complexity and stabilization of humus.

The temporal dynamics of the HA/FA ratio, a key indicator of compost humification, further supported the idea that diatomite enhances the humification process. Figure 2D shows that the HA/FA ratio in all treatment groups exhibited a consistent upward trend throughout the composting process. Initially, the HA/FA ratios for all groups was 0.91. By day 9, both DE-amended treatments had exceeded the humification benchmark (1.6), peaking at 1.75 (DE4) and 1.99 (DE8), while the CK remained lower at 1.46. At the final maturity stage, the HA/FA ratios reached 1.84, 2.30, and 2.81, in the CK, DE4, and DE8, respectively. These findings suggest that diatomite addition significantly enhanced the HA/FA ratio, thereby improving the degree of humification and potentially shortening the humification cycle, thus accelerating compost maturity.

### 3.3. Effect of Diatomite on Fungal Community Dynamics

#### 3.3.1. Effect of Diatomite on Fungal Community Diversity

**Figure 3:**
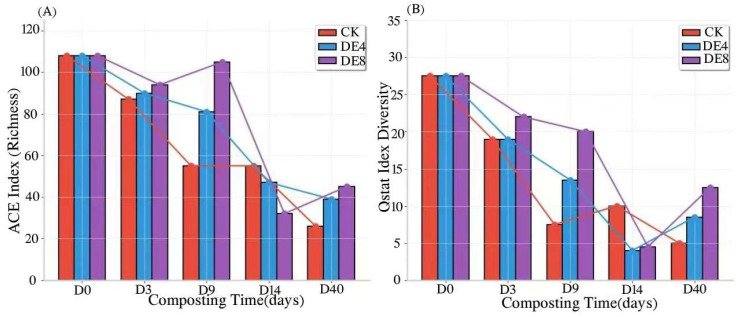
The temporal dynamics of fungal alpha diversity during composting. (**A**) ACE richness index and (**B**) Qstat diversity index across different sampling stages (day 0 to day 40). The X-axis represents composting time (days), and the Y-axis represents the calculated index values. The treatment color codes are as follows: red bars indicate the control treatment (CK), blue bars indicate the treatment with 4% (*w*/*w*) diatomite addition (DE4), and purple bars indicate the treatment with 8% (*w*/*w*) diatomite addition (DE8).

The ACE and Qstat indices reflect the richness and diversity of the fungal community, respectively. Figure 3A,B show that the CK exhibited continuously decreasing richness and a fluctuating downward trend in diversity throughout composting. In contrast, DE8 presents a distinctly different trend of change. During the early thermophilic phase (D3), the decrease in richness and diversity is markedly smaller relative to the CK. The richness recovery and superior diversity in DE8 by the end of the thermophilic phase (day 9) indicate this stage (days 3–9) might be a key interval for diatomite’s influence on the fungal community. Diatomite likely improves the microenvironment through its porous structure, which facilitates moisture adsorption, temperature regulation, and pH buffering, thereby potentially counteracting the heat-induced inhibition of the fungal community. By the maturation phase (D40), the richness and diversity of DE8 are approximately 1.8 and 2.6 times higher than those of the CK, respectively. These results indicate that 8% diatomite treatment effectively contributed to maintaining fungal community richness and diversity by promoting thermophilic fungal succession, potentially ensuring community stability throughout the composting process.

#### 3.3.2. Effect of Diatomite on Fungal Community Structure

**Figure 4:**
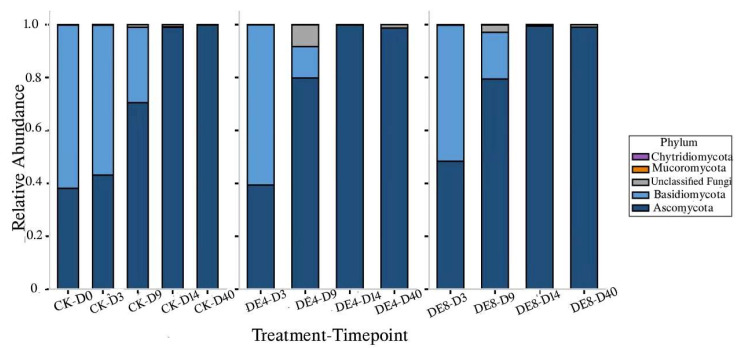
Succession of fungal community composition at the phylum level during composting. The stacked bar chart illustrates the relative abundance (represented as a fraction of 1.0 on the Y-axis) of major fungal phyla across five sampling stages: D0 (day 0), D3 (day 3), D9 (day 9), D14 (day 14), and D40 (day 40). The treatment groups in the experiment are as follows: CK (control, without additives), DE4 (amended with 4% *w*/*w* diatomite), and DE8 (amended with 8% *w*/*w* diatomite). The treatment color codes in the legend correspond to specific fungal phyla, with dark blue representing Ascomycota, light blue indicating Basidiomycota, and grey denoting unclassified fungi. Due to very low relative abundances, some taxa shown in the legend may not be visibly distinguishable in the stacked bars; all listed taxa were detected in the samples.

During composting, the fungal community exhibited distinct phylum-level successional characteristics (Figure 4). In the initial materials, Basidiomycota was more prevalent than Ascomycota. As composting progressed, the numbers of Ascomycota continuously increased in all treatments, ultimately exceeding 98% at D40, while the number of Basidiomycota declined sharply to below 0.04% across all treatments. This successional trend can largely be attributed to the significant differences in thermal tolerance between the two phyla. Previous studies indicate that Ascomycota maintains high activity at 60 °C, and this thermotolerance may confer a significant ecological advantage in high-temperature environments, enabling Ascomycota to play important roles in lignocellulose degradation [25,26]. Notably, the abundance of Basidiomycota in DE4 (11.9%) and DE8 (17.6%) was significantly lower than in the CK (28.5%) by day 9, suggesting that diatomite addition potentially accelerated its decline while enabling thermotolerant Ascomycota to achieve dominance more rapidly. Diatomite addition appeared to accelerate the heat-induced decline in the quantity of Basidiomycota, leading to its near-total disappearance by day 14 and thus diverging from the conventional successional trajectory of late-stage Basidiomycota dominance [27]. These results suggest that diatomite might improve composting community structure by promoting the early dominance of thermotolerant Ascomycota, thereby enhancing composting maturation efficiency.

**Figure 5:**
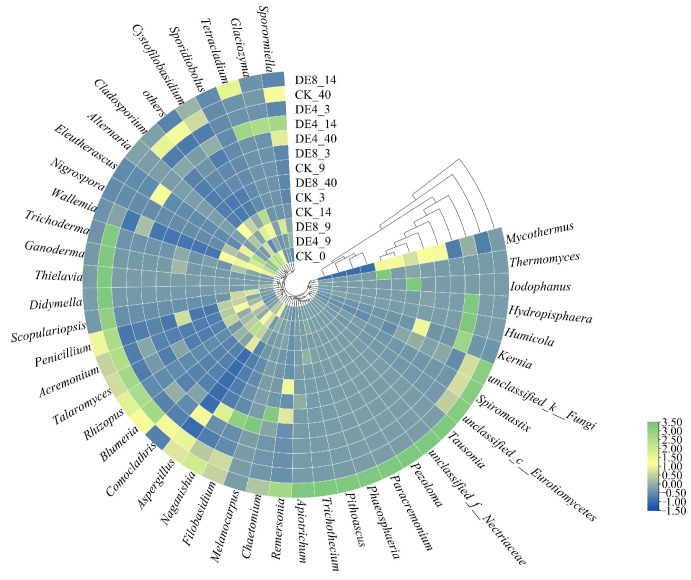
Circular heatmap illustrating the dynamic succession of the top 45 fungal genera during a 40-day aerobic composting process. The experimental setup included a control group (CK, pig manure and wheat straw) and two diatomite-amended treatments: 4% *w*/*w* diatomite (DE4) and 8% *w*/*w* diatomite (DE8). Samples were collected at five strategic time points: D0, D3, D9, D14, and D40. Color intensity corresponds to Z-score-normalized relative abundance values (unitless), ranging from blue (−1.50, low abundance) to green (3.50, high abundance). Hierarchical clustering was performed based on Bray–Curtis distance. Data are presented as the mean of three independent biological replicates (*n* = 3) for each treatment per sampling stage.

Heatmap analysis of 45 dominant fungal genera (Figure 5) indicated that diatomite addition notably altered the successional trajectory of fungal communities at the genus level. During the early thermophilic phase (D3), the thermophilic ascomycete *Mycothermus* dominated absolutely. Concurrently, the abundance of the halotolerant genus *Wallemia*, which initially accounted for 61.37% of the community, declined precipitously to less than 0.05% across all treatments, reflecting its high sensitivity to elevated temperatures. From the late thermophilic phase to the cooling phase (D9–D14), *Aspergillus* succeeded as the core functional genus. Diatomite addition appeared to expedite its colonization, with abundances in DE4 (42.4%) and DE8 (38.2%) peaking as early as day 9, whereas the CK reached its maximum (47.6%) later on day 14. These findings suggest that while diatomite may have advanced the colonization timeline, the CK ultimately achieved a higher peak abundance. Additionally, the thermophilic fungus *Mycothermus* exhibited stronger recovery potential in the DE treatments (especially DE8), with its abundance at D14 being significantly higher in DE8 (31.1%) and DE4 (10.3%) compared with the CK (<0.01%). As composting transitioned into the maturation phase (day 40), the fungal community structure underwent a substantial successional shift. In DE8, *Mycothermus* abundance significantly recovered to 99.6%, while DE4 also reached 87.1%, whereas the CK declined to 12.3%. Meanwhile, *Aspergillus* abundance in the CK increased to 64.3%, whereas in DE4 and DE8, it decreased to 0.45% and 0.20%, respectively. These results demonstrate that 8% diatomite may effectively optimize the fungal community structure in the late composting stage by accelerating functional fungal succession and promoting thermophilic fungal recovery.

### 3.4. Fungal Co-Occurrence Patterns

**Figure 6:**
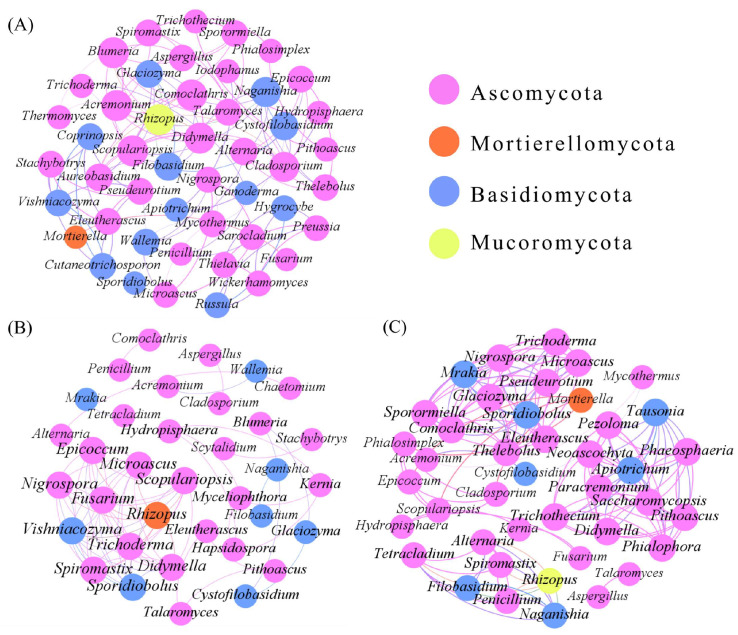
Fungal co-occurrence network analysis during composting. (**A**) CK treatment (control), (**B**) DE4 treatment (4% diatomite), and (**C**) DE8 treatment (8% diatomite). Node colors represent different fungal phyla: pink, orange, blue and yellow indicate Ascomycota, Mortierellomycota, Basidiomycota, and Mucoromycot, respectively. The networks were constructed based on Spearman correlation analysis of the relative abundance of fungal genera. The statistical notation for the edges includes a strict screening threshold of |r| > 0.6 and *p* < 0.05 to ensure the identified biotic interactions are robust. Each node represents a fungal genus, and its size is proportional to its degree of connectivity within the microbial ecosystem.

To systematically elucidate potential interspecific interactions and the community structural stability of key taxonomic units in fungal communities and analyze differences in fungal co-occurrence patterns under different treatment conditions, we conducted network analysis on fungal samples subjected to different treatments. By screening taxonomic units with significant correlations (*p* < 0.05, r > 0.6), a fungal co-occurrence network map was constructed to investigate the effects of diatomite addition on fungal symbiotic patterns. Figure 6A shows that the interspecific associations between Ascomycota, Basidiomycota, Mortierellomycota, and Mucoromycota in the CK exhibited significant positive correlations, suggesting that these taxa tend to form functionally cooperative symbiotic clusters during composting.

Figure 6B,C show that the network complexity of the two treatment groups (DE4 and DE8) exposed to diatomite exhibited an upward trend compared with the CK. Regarding network modularity, the co-occurrence networks of all treatments exhibited values higher than 0.4, indicating that all the fungal communities maintained a baseline modular structure with potential niche differentiation. Fungal groups in different functional classes tended to aggregate and form tightly interconnected modules. Regarding species interaction correlations, all three treatment groups exhibited a positively correlated dominance pattern, indicating that compost fungal community interactions primarily correspond to a cooperative symbiosis model. From the perspective of core functional fungi, the CK (control group) primarily relied on the synergistic action of Ascomycota and Basidiomycota to initiate organic-matter decomposition. In DE4, Ascomycota appeared to be the key taxon associated with enhancing the rates at which cellulose and hemicellulose degrade, thereby accumulating abundant precursor substances for subsequent humification. DE8 exhibited a trend of strengthening the function of Basidiomycota, with its secreted lignin-degrading enzymes potentially working synergistically to accelerate the deep transformation of recalcitrant substances such as lignin. The results of fungal co-occurrence network analysis indicated that diatomite addition had no significant impacts on the species richness (node number N) of the compost fungal community. Although the node numbers showed minor fluctuations in the control group (CK, N = 48), 4% diatomite group (DE4, N = 35), and 8% diatomite group (DE8, N = 42), no significant differences were observed overall.

Diatomite exhibited notable regulatory effects on the intensity of fungal interactions (edge count E) and symbiotic patterns. Notably, the DE8 group demonstrated significantly higher edge counts (E = 148) compared with the CK group (E = 117) and DE4 group (E = 74), suggesting richer network connectivity. DE8 exhibited significantly higher graph density (0.172) and average degree (7.048) values than the CK (0.104 and 4.875) and DE4 (0.124 and 4.229). Conversely, its modularity (0.696) fell slightly below that of the CK (0.752) but exceeded DE4 (0.436). This trade-off demonstrates that DE8 stability relies on global connectivity and network complexity rather than modularity. The high modularity in the CK reflects localized niche isolation due to unimproved conditions. Conversely, the 8% diatomite amendment provided an optimized porous matrix that stimulated cross-module cooperation, which mathematically lowered modularity boundaries while fundamentally driving the accelerated transformation of organic matter. Diatomite amendment significantly enhanced the connectivity and proportion of positive correlations within the fungal co-occurrence networks, with the most substantial impact observed in DE8. This group significantly promoted positive interactions between dominant functional fungi while maintaining the stability of species diversity, thereby providing a potential ecological foundation for lignin degradation and humification. In contrast, the addition of 4% diatomite slightly increased species diversity but led to a significant reduction in the intensity of fungal interactions.

Considering the results of this study, we suggest that differences in fungal interactions mediated by different diatomite addition ratios could influence the functional performance of key degrading taxa. Previous studies have shown that *Rhizopus* can effectively decompose lignin by secreting lignin-degrading enzyme systems (e.g., lignin peroxidase and laccase) [28]. *Mycothermus* helps advance compost maturation via spatial optimization and enzymatic degradation. On the one hand, it permeates the interior of a material to widen the enzymes-substrate contact area, effectively triggering the decomposition of organic-matter [29]. On the other hand, it exerts competitive dominance in the microenvironment by secreting thermostable cellulase and ligninase to thoroughly transform recalcitrant lignocellulose fractions [30]. In this study, under the environment of strong positive fungal correlations in DE8, *Rhizopus* and *Mycothermus* were more likely to form a synergistic degradation relationship with niche complementarity, which helps improve the fungal community’s overall organic-matter conversion efficiency. In contrast, DE4 exhibited weak fungal interactions, potentially leading to loose cooperation between key degrading fungi and insufficient functional coupling, resulting in lower overall degradation and transformation efficiency relative to DE8. In summary, the addition of diatomite reshaped the interactions between fungi during composting, thereby increasing the humification of the compost.

### 3.5. Relationships Between Composting Factors

**Figure 7:**
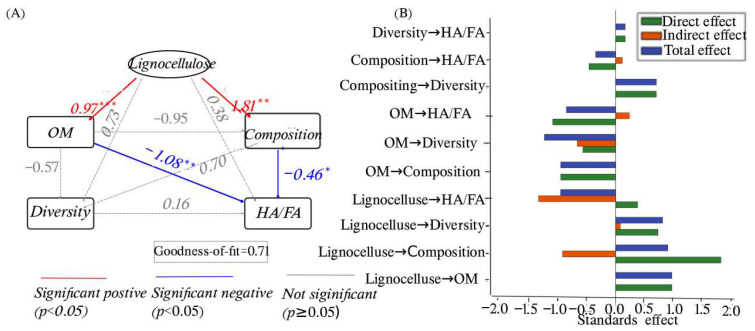
Partial least squares path modeling (PLS-PM) elucidating the mechanistic drivers of humification during aerobic composting amended with diatomite. (**A**) Path diagram illustrating the causal relationships between lignocellulose, organic matter (OM), fungal community composition, diversity, and the degree of humification (HA/FA ratio). The numbers on the arrows represent standardized path coefficients Solid red lines indicate significant positive relationships (*p* < 0.05), solid blue lines indicate significant negative relationships (*p* < 0.05), and dashed gray lines indicate non-significant relationships (*p* ≥ 0.05). (**B**) Standardized effects (direct, indirect, and total) derived from the PLS-PM. The X-axis represents the magnitude of the standardized effect (ranging from −2.0 to 2.0). The green, origin, and blue bars represent direct, indirect effects, and total effects, respectively. Statistical notation: * *p* < 0.05, ** *p* < 0.01, and *** *p* < 0.001. The Goodness of Fit (GoF) index for the model is 0.71.

To evaluate the potential relationships within the diatomite-amended composting system, a PLS-PM was developed and validated using the plspm package in R. The model’s reliability was confirmed by a Goodness of Fit (GoF) index of 0.71, and all latent constructs met the internal consistency requirements, with outer loadings exceeding 0.7. The statistical significance of the path coefficients was determined through bootstrapping with 1000 resamples. PLS-PM analysis was further employed to elucidate the potential impacts of organic fractions and fungal community characteristics on the degree of humification within the diatomite-amended composting system (Figure 7A,B). To ensure the model’s interpretability, each latent construct was defined by specific manifest variables: lignocellulose (cellulose, hemicellulose, and lignin content), OM (organic matter content), composition (PCoA axis 1 scores based on the relative abundance of the fungal phylum), diversity (Shannon and ACE indices), and HA/FA HA/FA ratio). The results showed that OM content exhibited a significant negative correlation with HA/FA ratio (path coefficient = −1.08, *p* < 0.01; total effect = −0.849). This inverse relationship suggests that as composting progressed, organic matter was continuously degraded via fungal activity, with its decomposition products serving as essential precursors for HA synthesis, thereby increasing the HA/FA ratio and the degree of humification. Within this diatomite-optimized system, the effect of lignocellulose on HA/FA was primarily mediated through indirect pathways (total effect = −0.950), whereas its direct effect was not statistically significant (0.378, *p* > 0.05). The total indirect negative effect of lignocellulose on HA/FA (−1.328) was mainly realized by promoting OM transformation (path coefficient = 0.966) and modulating fungal composition (path coefficient = 1.81). Specifically, the breakdown of lignocellulose provided carbon sources that sustained fungal biomass and metabolic activity, in turn increasing the organic precursor pool and shifting community composition, indirectly steering the direction of humification [31]. Additionally, community composition exerted a significant direct negative effect on HA/FA (−0.460, *p* < 0.05), suggesting that fungal succession contributed to humification by enhancing organic-matter degradation efficiency. Notably, lignocellulose exhibited a strong positive driving effect on composition (1.81, *p* < 0.01), while OM showed a negative regulatory trend with respect to composition (−0.946, *p* = 0.063), indicating their divergent roles in shaping the fungal community. In contrast, the effect of diversity on HA/FA was weak and not significant (0.163, *p* > 0.05), indicating that community diversity was not a key factor driving humification in this study. In conclusion, these findings suggest that diatomite addition enhanced the composting process by regulating lignocellulose degradation dynamics and organic-matter transformation rates. This process was associated with fungal community succession toward superior humification efficiency, ultimately promoting the formation of humic-acid-rich mature compost.

## 4. Discussion

### 4.1. Diatomite-Driven Lignocellulose Degradation and Its Contribution to Deep Humification

The addition of diatomite significantly accelerated the degradation and humification of organic components during pig manure–wheat straw composting, with the 8% amendment yielding the most pronounced effect. This finding directly validates our central hypothesis that diatomite is closely associated with efficient lignocellulose degradation and deep humification via microenvironmental optimization. It also directly addresses the central question raised in the Introduction: how might diatomite promote efficient lignocellulose degradation, and thus, enhance compost humification? Our experimental data show that the addition of 8% diatomite increased the degradation rates for organic matter, lignin, cellulose, and hemicellulose by 9.05%, 9.27%, and notable margins, respectively. This finding aligns with the results reported by Zhang [32], who found that the combined application of biochar and microorganisms can extend the thermophilic phase and enhance lignocellulose degradation. Notably, the majority of lignin degradation occurred during the later stages of composting, indicating that diatomite exerts a sustained and delayed promotive effect on the degradation of recalcitrant components. This result aligns with the findings made by Ren [33], who reported that diatomite improves aeration and water retention through its porous structure. This study further quantifies diatomite’s contribution to lignocellulose degradation.

Humic substances (HSs) are essential products of composting, and the dynamic transformation between the components HA and fulvic acid (FA) serves as a core indicator of the humification process [34,35]. Generally, an HA/FA ratio exceeding 1.6 indicates structural stabilization and a marked improvement in the quality of humic substances. In the CK treatment, the HA/FA ratio increased slowly during the early composting stage, indicating a delayed humification process characterized by limited HA accumulation and delayed FA degradation. In contrast, the addition of diatomite was closely associated with an increased abundance of potential humification-related microorganisms, as it adsorbed organic matter and improved the fungal microenvironment through its porous structure. This process was accompanied by HA formation and FA conversion, resulting in higher total HS content, an enriched HA fraction, and a significantly elevated HA/FA ratio. Under the DE8 treatment, the degree of humification exhibited the most pronounced increase during the late composting stage, with the HA/FA ratio surpassing 1.6. This finding indicates that a high dosage of diatomite exerts the most stable and pronounced promotive effect on compost humification. It also corroborates Bai’s [36] supposition that prolonging the thermophilic phase can enhance the humification process (Table 1), deepening our understanding of the mechanism by which diatomite regulates compost humification.

### 4.2. The Bridging Role of Fungal Community Succession in the Transformation of Refractory Components and Humus Synthesis

This study reveals the fungal mechanism by which diatomite is associated with enhanced lignocellulose degradation and humification through the regulation of fungal community succession. The key factors underlying this effect are the optimization of fungal community structure and the enhancement of core functional groups. Within Ascomycota, thermophilic genera such as *Mycothermus* and *Aspergillus* exhibited significantly enhanced colonization during the thermophilic phase and greater recovery in subsequent stages. Studies on humic acid urea have also identified *Aspergillus* as a key fungal genus for straw decomposition [37]. The accelerated decline of Basidiomycota observed in the diatomite treatments reflects a rapid niche substitution driven by species-level functional redundancy. The optimized microenvironment and faster heat accumulation suppressed the mostly mesophilic Basidiomycota. However, their lignocellulose-degrading roles were effectively compensated for by the synchronized enrichment of these thermotolerant Ascomycota functional groups. This functional redundancy maintains the continuous decomposition of the cellulose and lignin skeleton, thereby ensuring the smooth progress of the humification process. By stabilizing micro-environmental moisture and pH through its porous structure, diatomite alleviates high-temperature stress and provides a favorable ecological niche for thermotolerant fungi, potentially maintaining their metabolic potential activity and ensuring community continuity. These observations are consistent with the research conducted by Tian [25] and further supports the central role of fungi in organic-matter conversion during composting. In addition, co-occurrence network analysis revealed that diatomite shifts fungal interactions from competition-driven dynamics to cooperative symbiosis by optimizing the compost microenvironment. Compared with the CK, DE8 exhibited increased network complexity, forming a highly connected functional module centered on *Mycothermus*. This shift from competition to cooperation was strongly correlated with community stability and suggested a greater potential for high material transformation efficiency. Furthermore, diatomite reshaped the successional trajectory of the fungal community, driving a transition from a rapid decomposition phase in the early composting stage to an advanced transformation phase during the thermophilic stage and finally a stable decomposition phase at the maturation stage. This successional shift was strongly correlated with higher carbon and nitrogen decomposition efficiency and enhanced community stability, ultimately leading to better overall compost quality. These findings corroborate those reported by Mehta [17] regarding the ability of thermophilic fungi to synergistically degrade organic matter. Consistently, enhanced fungal network complexity and synergistic interactions have been shown to improve composting efficiency [38,39]. In agricultural soils, organic amendments can increase the proportion of positively correlated microorganisms, thereby reducing fungal competition over nutrients [40]. Furthermore, Zhang [32] further indicated that strengthening microbial collaboration within fungal networks significantly increases the humus content.

PLS-PM analysis further elucidated the statistical relationships between fungal communities in regard to regulating humification. Prior to path interpretation, the structural integrity of the outer model was rigorously validated; the multi-indicator blocks (lignocellulose and diversity) exhibited satisfactory convergent validity, with Average Variance Extracted (AVE) values exceeding 0.50 and a Composite Reliability (CR) above 0.70, while single-indicator blocks held fixed parameters, confirming the reliability of the measurement framework. Organic matter (OM) showed a significant negative correlation with the HA/FA ratio (path coefficient = −1.08, *p* < 0.01), indicating that its degradation products serve as precursors for HA synthesis. Fungal community succession was identified as a key potential predictor governing humic acid maturation [40]. Lignocellulose indirectly regulated humification by promoting OM accumulation (path coefficient = 0.966) and modulating fungal community composition (path coefficient = 1.81). In previous studies, fungal inoculation was shown to promote humus formation through the secretion of extracellular enzymes such as cellulase and lignin peroxidase [41]. In our study, however, because direct measurements of enzyme activities were not conducted, this physiological pathway cannot be directly proven. Instead, the strong path coefficient between lignocellulose and fungal community composition in our model captures a structural shift at the taxonomic level. This robust statistical correlation suggests that the diatomite amendment optimized the composting microenvironment to favor the enrichment of specific fungal genera, which are widely documented to possess these functional degradation traits. Notably, organic matter exerted a negative, albeit non-significant, regulatory trend with respect to community composition (−0.946, *p* = 0.063), partially offsetting the strong positive effect of lignocellulose (1.81, *p* < 0.01). Mathematically, the path coefficient exceeding 1.0 (1.81) represents a “suppressor effect” triggered by high multicollinearity (VIF = 14.9) between lignocellulose and OM [42]. This reflects an ecological reality where the direct promotive effect of lignocellulose with respect to fungal succession is counterbalanced by negative feedback from OM consumption, yielding a stable and valid total effect of 0.897. Consequently, while microbial community composition is generally recognized as a major descriptor for compost humification [43], the critical role of fungal succession observed here should be interpreted as a potential statistical predictor rather than direct functional causality. In contrast, community diversity had no significant effect on the HA/FA ratio (0.163, *p* > 0.05), indicating that humification is driven by key functional groups rather than by overall species richness. Compost fungal network analysis further indicated that functional groups serve as stronger predictors of humification dynamics than taxonomic groups [44]. Taken together, these results, from both network topology and structural equation modeling, firmly support the biological dimension of our hypothesis, demonstrating that the structural reshaping and cooperative succession of the fungal community, as opposed to simply taxonomic diversity, acts as a key potential driver of advanced humification. This study advances our understanding of how mineral additives regulate fungal functional pathways during composting, underscoring the indispensable role of fungi in the advanced transformation of organic matter. However, this lab-scale study is limited to a single pig manure–wheat straw system. Quantitative results, such as humification time cycles and fungal succession changes, are system-specific and may vary under industrial conditions or changes in compost material composition [45,46]. Nevertheless, our findings remain informative. The mechanism whereby diatomite improves the microenvironment to shift the fungal community from competition to cooperation provides a useful framework, offering valuable guidance for other composting systems facing slow-degradation challenges. To address the limitations of relying purely on taxonomic inference, future research must integrate direct enzyme activity assays and quantitative PCR of key functional genes, as well as metatranscriptomic and metabolomic approaches to elucidate the active expression and metabolic regulation mechanisms of key functional fungal communities, thereby providing a theoretical foundation for the efficient resource utilization of agricultural waste.

## 5. Conclusions

In summary, this study firmly validates our hypothesis that an 8% diatomite amendment represents the optimal dosage for maximizing compost humification. This treatment significantly accelerated lignocellulose bioconversion, achieving cumulative degradation rates of 63.52% for cellulose and 75.22% for hemicellulose throughout the composting process, alongside a 13.72% lignin degradation rate specifically during the maturation phase. Notably, this treatment distinctly elevated the HA/FA ratio and advanced the final compost humification degree. Mechanistically, co-occurrence network analysis revealed that the amendment reshaped fungal interactions from localized niche isolation into global, cross-module cooperation (evidenced by higher graph density and edge counts). This framework fostered synergistic coupling between key degrading taxa (*Rhizopus* and *Mycothermus*), mathematically compressing modularity boundaries to facilitate recalcitrant matter conversion. Ultimately, partial least squares path modeling indicated that fungal community composition played a more pivotal role than alpha diversity in modulating this organic-component transformation process, thereby crucially contributing to the microbially mediated humification process.

## Figures and Tables

**Table 1:** Variations in temperature during the composting process.

Treatment	Average High Temperature	High-Temperature Periods	Temperature			
			(Day 3)	(Day 9)	(Day 14)	(Day 40)
CK	58.8	9	56.5	50.0	39.0	19.0
T1	63.2	10	57.9	53.0	45.2	20.0
T2	64.5	10	59.5	55.0	47.0	21.5

## Data Availability

The raw data supporting the conclusions of this article will be made available by the authors on request.

## Abbreviations

The following abbreviations are used in this manuscript:

PLS-PM	Partial least squares path model
HA	Humic acid
FA	Fulvic acid
HS	Humic substance
OM	Organic matter
